# Editorial: Next Therapeutic Targets in Ocular Diseases

**DOI:** 10.3389/fmed.2022.953377

**Published:** 2022-06-23

**Authors:** Ryoji Yanai, Yoko Okunuki, Dong Ho Park, Embong Zunaina

**Affiliations:** ^1^Department of Ophthalmology, Yamaguchi University Graduate School of Medicine, Yamaguchi, Japan; ^2^Janssen Biopharma, South San Francisco, CA, United States; ^3^Department of Ophthalmology, School of Medicine, Kyungpook National University, Kyungpook National University Hospital, Daegu, South Korea; ^4^Cell and Matrix Research Institute, Kyungpook National University, Daegu, South Korea; ^5^Department of Ophthalmology and Visual Science, School of Medical Sciences, Universiti Sains Malaysia, Kubang Kerian, Malaysia

**Keywords:** diabetic macular edema, glaucoma, uveitis, pediatric cataract, inherited retinal diseases, age-related macular degeneration, digitally assisted vitreoretinal surgery, 27-gauge vitrectomy

Vision impairment significantly impacts the length and quality of life (QOL). Over the last several decades, there has been a revolution in our understanding of ocular diseases and an advance in the development of medical and surgical treatment for patients. Particularly in developed countries, the leading causes of vision impairment are diabetic retinopathy, glaucoma, and age-related macular degeneration (AMD).

The most recent medical treatment in ocular diseases is anti-vascular endothelial growth factor (VEGF) therapy for AMD, diabetic macular edema (DME), and neovascular glaucoma. However, the currently available treatments are ineffective for some ocular diseases. These inefficacies in the treatment of vision impairments need to be addressed.

Given these unmet medical needs, it is imperative to investigate the pathological factors that constitute the risks of vision impairment. It is this important topic that provides a platform for this collection of papers to explore the next therapeutic targets in ocular diseases with visual impairment. The inquiry can be sub-divided into three categories:

1. Cutting-edge treatments for DME and diabetic retinopathy: VEGF and next related factors.
2. Next medical applications of treatment options for ocular diseases: gene therapy, new or existing anti-inflammatory therapy.
3. Advanced surgical technologies for ocular diseases: exploring novel usages of these medical devices, and also the application of VEGF.

The first category, *Cutting*-edge *treatments for DME and diabetic retinopathy*, involves the selective accumulation of clinical studies in patients with DME, or diabetic retinopathy, in order to investigate future therapeutic options. As mentioned in the introduction section, anti-VEGF agents such as ranibizumab are the most successful treatment options for DME, which are pathologically linked to the disruption of the blood retinal barrier broken by VEGF. Nonetheless, monthly injections of ranibizumab are impractical as the cost of anti-VEGF agents and the requirement of frequent clinic visits can be serious barriers to patient compliance to the treatment regimen. Lai et al. demonstrated that a treat-and-extend (T&E) regimen with ranibizumab at 4-week intervals effectively improved best corrected visual acuity (BCVA) and reduced central retinal thickness (CRT) for 91 eyes from the 64 patients they studied. In their contribution, Chen et al. found that erythropoietin has an angiogenic potential equal to VEGF, and that intravitreal injection of ranibizumab (IVR) discernibly reduced the erythropoietin level, but not enough to the normal level when they compared 24 proliferative diabetic retinopathy patients with 11 non-diabetic patients.

To determine the prognostic factors in vitrectomized eyes with DME, which is not the effect of anti-VEGF therapy, Liang et al. demonstrated that intraocular inflammation has an important influence on the pathogenesis of DME in 36 vitrectomized eyes when compared with 71 treatment-naïve eyes and suggested that anti-inflammatory therapies may represent another strategy for the treatment of DME in vitrectomized eyes. Moreover, Hsia et al. evaluated the effectiveness and safety of anti-inflammatory therapies as intravitreal dexamethasone (DEX) implants in refractory DME treated by intravitreal ranibizumab. They concluded that switching to a DEX implant is not only feasible but also safe for treating patients of DME refractory with intravitreal ranibizumab.

In an insightful work using acrolein, a highly reactive aldehyde that covalently binds to cellular macromolecules, Fukutsu et al. demonstrated that Rho-associated coiled-coil-containing protein kinases-1 (ROCK-1) mediated the migration of retinal glial cells: a pathological hallmark of diabetic retinopathy. Although VEGF is one of the prominent participants in the pathological factors in diabetic ocular disease, the current data suggests that inflammation is the next therapeutic target in the mechanisms of progressive and encouraging factors in DME and diabetic retinopathy.

The second category of papers in this collection, *Medical applications of ocular diseases*, explores the next medical treatment options, including gene therapy, biologics, and immune mediators. Inherited retinal dystrophies represent a clinical and Research Topic of great interest. This is because of the lack of approved treatments in most cases and also because of the increasing prevalence related to the evolution of diagnostic approaches to detect these diseases early. In their paper, Amato et al. reviewed the current state-of-the-art, and the rapidly evolving future perspectives regarding gene therapy in the primary inherited retinal dystrophies in order to provide an updated, broad, and comprehensive scenario regarding the present situation and future attitudes.

As is now widely known, and generally accepted, uveitis is one of the significant causes of vision loss and is estimated to cause ~10% blindness in developed countries. Immunosuppressive therapy, including local or systemic corticosteroids, is the primary treatment for non-infectious uveitis. Although prolonged corticosteroid used leads to severe ocular and systemic side effects, it is now increasingly hoped corticosteroid-sparing agents will be the next effective and practical therapeutic agents in ocular inflammatory diseases. Harada et al. performed clinical studies and found the efficacy and safety of methotrexate in treating Japanese patients with non-infectious uveitis. Hiyama et al. also investigated immunosuppressive therapies' clinical characteristics and efficacy in 65 eyes in 35 patients (14 male and 21 female) with Vogt-Koyanagi-Harada (VKH) disease. They proposed the treatment possibility for patients with late-stage VKH disease with adalimumab and low-dose methotrexate combination therapy. However, some severe uveitis cases are resistant to steroid treatment, multiple conventional disease-modifying antirheumatic drugs (methotrexate and salazosulfapyridine), and tumor necrosis factor-α (TNF-α) inhibitors (adalimumab and infliximab). Kaneko et al. reported the possibility of a Janus kinase (JAK) inhibitor, including baricitinib, as a viable option in treating uveitis with resistance to conventional treatment.

Some other medical procedures that can be categorized as *Medical applications for ocular diseases* are the effect of Sulforaphane (SFN), a natural isothiocyanate, reported by Sim et al. SFN effectively alleviates PM_2.5_-induced oxidative damage in human ARPE-19 cells by its antioxidant effects; additionally, SFN can potentially be used as a therapeutic agent for AMD, particularly in cases related to PM_2.5_ exposure. The role of oxymatrine as a transforming growth factor-β (TGF-β*)* and TNF*-*α inhibitor is to retard the development and progression of an animal model of glaucoma, as has been proposed by Das et al. in their contribution.

The third category, *Surgical technologies for ocular diseases*, is related to research efforts for the development of advanced therapeutic techniques. For instance, Kim et al. presented the data on the effectiveness and safety profile of 27-gauge pars plana vitrectomy (PPV) for various vitreoretinal conditions associated with uveitis. This study proposes that most uveitis specialists anticipate using 27-gauge PPV because of its minimally invasive nature which suit uveitis eyes.

Melega et al. conducted a randomized clinical trial to compare nylon sutures to polyglactin sutures in pediatric cataract patients and demonstrated that polyglactin 10-0 sutures in pediatric patients' cataract surgeries are safe and result in fewer post-operative complications than non-absorbable nylon 10-0 sutures.

In their innovative study on vitrectomy, Park et al. demonstrated that the customized color settings available in the digitally assisted vitreoretinal surgery (DAVS) system enabled surgeons to lower the indocyanine green (ICG) concentration as much as 3-fold, which would be helpful in reducing the ICG toxicity. This is the first study that quantitatively measured the macular color contrast according to different color channels using the DAVS color settings.

In another exciting contribution that suggests an alternative treatment to both surgery and the conventional medical treatment for pterygium, Omar et al. proposed the possible use of intralesional anti-VEGF as the future modality of adjunctive therapy for pterygium surgery.

This compendium of papers on this Research Topic provides interesting, innovative and helpful insights that can surely contribute to the development of subsequent management and treatment regimens for vision loss from ocular diseases, including DME, diabetic retinopathy, inherited retinal dystrophies, uveitis, AMD, glaucoma and pterygium. This timely collection of articles pertinently emphasizes the importance of DME and anti-VEGF therapies in basic research and clinical settings. It presents not only some novel and creative methods of anti-VEGF therapy, gene therapy, anti-inflammatory therapy, and offers improvements in current treatment practices for vitrectomy and pterygium surgery but also questions, re-considers and re-interprets widely held assumptions in the light of these emerging therapeutic procedures, application options and advanced technologies for ocular diseases. However, one limitation of this collection is the paucity of evidence. This insufficiency may well be because of the number of subjects studied, or the size of the data. Also, in some instances, the specific mechanisms for the studies could be rendered clearer, or made more lucid, by further elaboration.

It is our expectation and hope that this collective inquiry in the form of research papers will raise the level of understanding about, and also sharpen the focus on the pathogenesis of ocular diseases, including genomic, molecular, cellular predisposition. We also hope that the contributions assembled here will play a significant catalytic role in the future development of next therapeutic options.

## Author Contributions

RY drafted the manuscript. YO, DP, and EZ critically proofread and edited the manuscript. All authors contributed to the article and approved the submitted version.

## Funding

DP is financially supported by the Basic Science Research Program of the National Research Foundation of Korea (NRF) funded by the Korean government (Ministry of Science and ICT) (2019R1A2C1084371) and also supported by the Ministry of Science and ICT (MSIT) Korea under the Information Technology Research Center (ITRC) Support Program (IITP-2022-2020-0-01808) supervised by the Institute of Information and Communications Technology Planning and Evaluation (IITP). This research was supported by Korea Drug Development Fund funded by Ministry of Science and ICT, Ministry of Trade, Industry and Energy, and Ministry of Health and Welfare (HN21C0923000021, Republic of Korea).

## Conflict of Interest

YO was employed by Janssen Biopharma. The remaining authors declare that the research was conducted in the absence of any commercial or financial relationships that could be construed as a potential conflict of interest.

## Publisher's Note

All claims expressed in this article are solely those of the authors and do not necessarily represent those of their affiliated organizations, or those of the publisher, the editors and the reviewers. Any product that may be evaluated in this article, or claim that may be made by its manufacturer, is not guaranteed or endorsed by the publisher.

